# Genome-Wide Identification and Evolutionary and Expression Analyses of the Cyclin B Gene Family in *Brassica napus*

**DOI:** 10.3390/plants13121709

**Published:** 2024-06-20

**Authors:** Mingyue Li, Minghao Zhang, Boyu Meng, Likai Miao, Yonghai Fan

**Affiliations:** 1 Integrative Science Center of Germplasm Creation in Western China (CHONGQING) Science City, College of Agronomy and Biotechnology, Southwest University, Beibei, Chongqing 400715, China; lmy1980234@email.swu.edu.cn (M.L.); 18311870271@163.com (M.Z.); beyul1340516624@email.swu.edu.cn (B.M.); mlk970907@email.swu.edu.cn (L.M.); 2 Hanhong College, Institute of Innovation and Entrepreneurship, Southwest University, Beibei, Chongqing 400715, China

**Keywords:** cyclin B, evolution, expression pattern, seed size, *Brassica napus*

## Abstract

Cyclin B (CYCB) is a regulatory subunit of cyclin-dependent kinase (CDK), the concentration of which fluctuates to regulate cell cycle progression. Extensive studies have been performed on cyclins in numerous species, yet the evolutionary relationships and biological functions of the *CYCB* family genes in *Brassica napus* remain unclear. In this study, we identified 299 *CYCB* genes in 11 *B*. *napus* accessions. Phylogenetic analysis suggests that *CYCB* genes could be divided into three subfamilies in angiosperms and that the *CYCB3* subfamily members may be a newer group that evolved in eudicots. The expansion of *BnaCYCB* genes underwent segmental duplication and purifying selection in genomes, and a number of drought-responsive and light-responsive *cis*-elements were found in their promoter regions. Additionally, expression analysis revealed that *BnaCYCBs* were strongly expressed in the developing seed and silique pericarp, as confirmed by the obviously reduced seed size of the mutant *cycb3;1* in *Arabidopsis thaliana* compared with Col-0. This study provides a comprehensive evolutionary analysis of *CYCB* genes as well as insight into the biological function of *CYCB* genes in *B*. *napus*.

## 1. Introduction

The eukaryotic cell division cycle, a highly regulated process involving gene transcription and protein translation and degradation, is driven by the activity of cyclin-dependent kinases (CDKs) and regulatory cyclins (CYCs), which function primarily during the S-phase and at the G1/S and G2/M transitions [1]. Cyclins were initially defined in marine invertebrates as proteins that were expressed at specific points and then subsequently degraded during the cell division cycle [2]. Plants possess more cyclins than yeasts and animals [3,4]. The number and classification of cyclins are different in plant genomes, which rely on the protein phases and the sequences of amino acid to activate CDK partners during cell cycles. In *Arabidopsis thaliana*, 49 cyclins were identified and classified into A, B, D, H, L, T, U, and SDS types [5]. However, 48 cyclin genes were identified and classified into A, B, D, F, SDS, H, L, T and P types in rice (*Oryza sativa*) [6]. At the G1/S transition, CDKA/CYCD complexes are phosphorylated by CDK-activating kinase (CAK) to inactivate retinoblastoma-related (RBR) that permits heterodimeric E2F/DP transcription factors to stimulate S-phase gene expression, resulting in cell proliferation [3,7]. Moreover, the products of cyclin A and cyclin B genes assemble with CDKB, and then are stimulated by CAK or inhibited by CKIs, to promote M-phase progression [8,9]. Previous studies have suggested that the rate of cell division, the number of dividing cells, and the duration of the cell proliferation phase control the cell number of organs and tissues, which further influence their sizes [10]. For example, different division patterns could alter the cell size of the outermost floral [11], and cell cycle regulation is essential for the seed size in *Arabidopsis* [12]. It was also reported that the endoreduplication cycle is a means to boost fruit size and control the plant growth rate [11].

Typical cyclin B contains four conserved-type domains: (1) cyclin-N, a necessary domain in CYCBs that contains the CDK-binding site; (2) cyclin-C, a less conserved domain; (3) D-box, a domain involved in protein proteolysis; (4) PEST, a marker for unstable proteins [5,12,13,14]. Previous studies have illustrated that the *CYCB* family genes function to regulate spindle and phragmoplast formation during the cell cycle [15], which can promote plant growth and seed development in *A. thaliana* [16,17]. In *O. sativa*, *CYCB2;2* promotes cell division to accelerate root growth without increasing the cell size [18]. Mariana et al. [19] found that *CYCB1;1*, *CYCB1;2*, and *CYCB1;3* are redundantly required for endosperm proliferation, with reduced growth and aborted seeds in *A. thaliana*. Manipulation of the cell cycle procession by regulating the expression of *CYCB1;1* and *CYCB2;2* may improve rice yield performance [20]. These data suggest that B-type cyclins play a predictable role in stimulating cell division and promoting tissue development.

The characterization and contribution of *CYCB* family genes have been well studied in *A. thaliana*, *O. sativa,* and *Brassica rapa* [5,6,21], whereas no research has focused on *Brassica napus*, one of the most important allopolyploid crops worldwide. Here, we identified *CYCB* genes across 11 *B*. *napus* accessions and 24 representative angiosperms. To better understand *CYCB* function in *B*. *napus*, we analyzed the characteristics, gene structure, collinearity, *cis*-acting elements, protein networks, and expression patterns in Darmor-*bzh* (Dar), as well as functional characterization in the homologous mutant *A. thaliana*. Our study provides a comprehensive evolutionary analysis of type-B cyclin genes and an understanding of *CYCB* functions in *B*. *napus*.

## 2. Results

### 2.1. Identification of CYCB Genes in B. napus

We identified 25 *CYCB* genes in the *B*. *napus* accession Dar (Table 1 and Table S1). Each *AtCYCB* corresponded to 2–4 *BnaCYCB* homologs. However, no homologs were found for *AtCYCB1;4*, *AtCYCB1;5*, or *AtCYCB2;5* in *B*. *napus*. The number of amino acid residues in BnaCYCB proteins ranged from 204 (BnaCYCB3;1b) to 681 (BnaCYCB1;2b), and most of them contained about 410 aa (Table 1). The relative molecular weights (MWs) of BnaCYCB proteins ranged from 23,927.39 to 76,106.19 Da, with an average of 48,142 Da. The isoelectric points (pIs) of BnaCYCB proteins were predicted to range from 4.81 (BnaCYCB2;2a) to 9.86 (BnaCYCB3;1d), with 10 members possessing a pI greater more than seven and 15 members possessing a pI less than seven (Table 1). No transmembrane helixes or signal peptides were found in the BnaCYCB proteins, and all BnaCYCB members were predicted to localize in the nucleus (Table 1).

To compare the copy-number variations in *CYCB* family genes in *B*. *napus* accessions, we further identified *CYCB* genes among 11 accessions in *B*. *napus* (see Methods). In total, we identified 299 *CYCB* family genes in the 11 *B*. *napus* accessions, most of which presented 20–30 *CYCBs*, but 50 *CYCBs* were identified in Ningyou (Table 2 and Table S2). Furthermore, *CYCB1;1*, *CYCB1;2*, *CYCB2;2*, and *CYCB2;3* had more copies in Ningyou, suggesting an expansion of these genes in the Ningyou genome. Moreover, no homolog was found for *AtCYCB2;5* in any accessions, and *CYCB2;4* was identified only in Dar and *CYCB3;1* was only identified in Dar and ZS11, suggesting the differences in copy-number variations among *B*. *napus* accessions. *CYCB1;2*, *CYCB1;3*, *CYCB2;1*, *CYCB2;2*, and *CYCB2;3* members were identified in all *B*. *napus* accessions, illustrating their important roles in *B*. *napus* (Table 2).

### 2.2. Phylogenetic Analysis of CYCB Proteins

**Figure 1:**
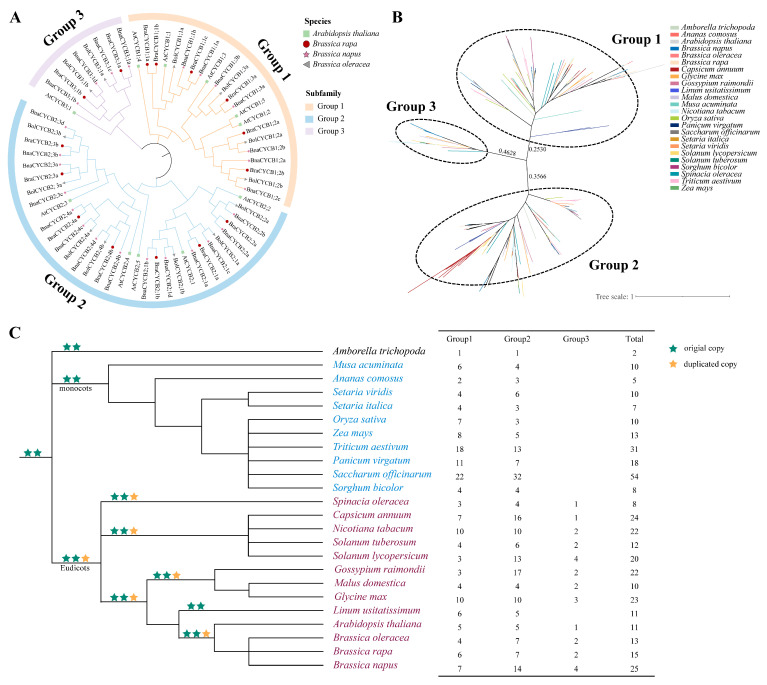
(**A**) Phylogenetic relationships among CYCB proteins in four Brassicaceae species; (**B**) phylogenetic relationships among CYCB proteins in 24 species; (**C**) number of *CYCB* genes in each group among 24 plant species. Phylogenetic relationships among these species refer to APG IV [22].

To reveal the evolutionary relationships among *CYCB* family genes in *A. thaliana*, *B*. *napus*, *B. rapa*, and *Brassica oleracea*, we constructed a neighbor-joining (NJ) tree using their protein sequences. The results showed that these CYCB proteins could be divided into three groups: Group 1 (CYCB1), Group 2 (CYCB2), and Group 3 (CYCB3). Group 2 was the largest, containing 33 Brassicaceae members, followed by Group 1 (22) and Group 3 (9) (Figure 1A, Table S1). The phylogenetic relationships among the homologous genes from these species were completely consistent with the relationships among the species. The CYCBs from *B*. *rapa* and *B*. *oleracea* first clustered with those from *B*. *napus* to form a small branch and then joined with CYCBs from *A. thaliana* to form a larger clade (Figure 1A). 

To further investigate the phylogenetic relationships of CYCB proteins in angiosperms, we also analyzed the phylogenetic relationships of CYCB family members in 24 species (see Methods). In total, 384 CYCB proteins were found in these examined plants (Table S2). The phylogenetic analysis suggested that these CYCB members could also be classified into three distinct groups (Figure 1B), corresponding to the above-mentioned Brassicaceae analyses. Specifically, Group 1 contained 159 CYCB members, Group 2 had 199 CYCB members, and Group 3 possessed 26 CYCB members. The copy-number of the CYCB members varied among these species from 2 (*Amborella trichopoda*) to 54 (*Saccharum officinarum*), with obvious expansion in Groups 1 and 2 but relative conservation in Group 3. Members of Groups 1 and 2 were examined in all monocot and eudicot plants, with one CYCB member in *A. trichopoda* (Figure 1C). Noticeably, CYCB members in Group 3 were detected only in eudicots (Figure 1C), suggesting that Group 3 may be a newer group in eudicot plants and may evolve from Group 1 or 2. However, we found that *Linum usitatissimum* did not contain CYCB3 members, suggesting that this copy may be lost in *L. usitatissimum*. In addition, the relationship among the CYCB members was in accordance with the evolutionary relationship of species. In each subfamily, CYCB members from Brassicaceae first clustered together to form a small clade with Solanaceae and then formed a larger eudicot cluster. Similarly, Poaceae plants clustered in a clade with *Musa acuminata* and *Ananas comosus* to form a monocot cluster and then clustered with eudicot cluster (Figure 1B).

### 2.3. Gene Structure and Protein Motif Analyses

**Figure 2:**
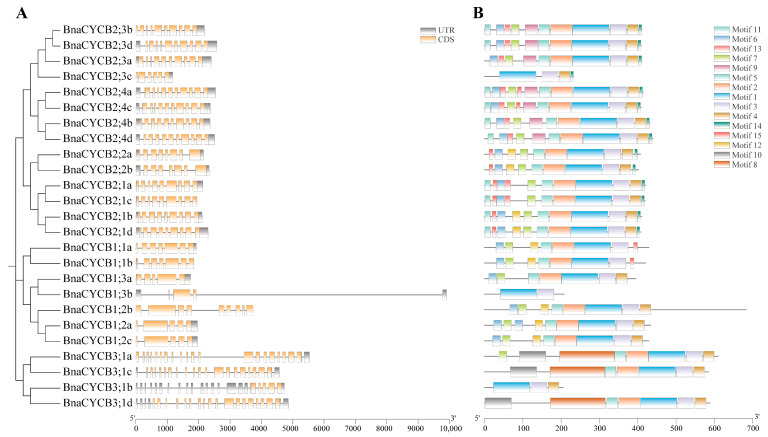
(**A**) Gene structures of *CYCB* genes and (**B**) conserved motifs.

To compare the gene structures of *BnaCYCB*, we aligned the coding sequences with their corresponding genomic sequences. The *BnaCYCB2* subfamily genes presented similar gene structures, with 8–10 introns (Figure 2A). Most genes in Group 3 were composed of 19–20 introns and 21–23 exons. Most Group 1 members contained 5–7 introns, with the exception of *BnaCYCB3;1b,* which had five exons, and *BnaCYCB1;3b,* which had two exons and the longest introns. Additionally, all *BnaCYCB* members contained at least one UTR structure except for *BnaCYCB1;2b* (Figure 2A).

Fifteen conserved motifs were identified in the full-length BnaCYCB proteins. Motifs 1 and 3 were observed in all 25 BnaCYCB proteins (Figure 2B), and most proteins contained motifs 2, 5, and 4. Motifs 2 and 5 were not found in BnaCYCB2;3c, BnaCYCB1;3b, or BnaCYCB3;1b, and motif 4 was not found in three Group 1 members (BnaCYCB1;1a, BnaCYCB1;1b, BnaCYCB1;3b) (Figure 2B). Motifs 9, 11, 13, and 14 were observed only in Group 2, and motifs 8 and 10 were observed only in Group 3. The BnaCYCB2 subfamily contained the most motifs, in contrast to the BnaCYCB3 subfamily with the least motifs, indicating a functional divergence in BnaCYCB subfamilies.

### 2.4. Chromosomal Locations and Collinearity Analysis

**Figure 3:**
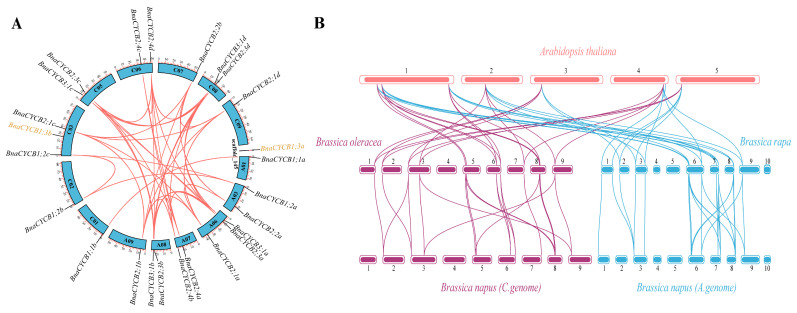
(**A**) Chromosomal locations and collinearity analysis of *CYCB* genes in *Brassica napus*. The black gene labels represent genes in the collinearity region, and the yellow gene labels represent genes without a collinearity relationship with *BnaCYCB*. (**B**) Syntenic relationships of *CYCB* genes in *Arabidopsis thaliana*, *Brassica oleracea*, *Brassica rapa,* and *Brassica napus*.

The 24 *BnaCYCB* genes were located on 14 chromosomes, and *BnaCYCB1;3a* was located on a scaffold, with 11 genes in the A subgenome and 13 in the C subgenome (Figure 3A). No *BnaCYCB* genes were found on chromosomes A02, A04, A05, A10, or C04. All chromosomes with *BnaCYCB* contained one or two genes, except A06 and C03 with three gene locations (Figure 3A). In *B*. *napus*, 40 pairs composed of 23 *CYCB* genes were predicted to share syntenic relationships, with tandem duplications found in *BnaCYCB2;4a*, *BnaCYCB2;4b*, *BnaCYCB2;4c*, and *BnaCYCB2;4d* (Figure 3A).

Furthermore, the collinearity map among Brassicaceae species showed a great number of homologous *CYCB* genes in *A. thaliana*, *B*. *oleracea*, *B*. *rapa,* and *B*. *napus*. Seven and eight *AtCYCBs* shared syntenic relationships with 12 *BolCYCBs* and 13 *BraCYCBs,* respectively, including six to seven multi-copy genes and one single-copy gene (*AtCYCB1;1*) (Figure 3B). No homolog for *AtCYCB2;3*, *AtCYCB1;5*, or *AtCYCB1;4* was found in either *B*. *oleracea* or *B*. *rapa*, indicating that genes losses occurred in *B*. *oleracea* and *B*. *rapa* during evolution. The *CYCB* members from diploids *B*. *oleracea* and *B*. *rapa* mostly overlapped with those from the C and A subgenomes of *B*. *napus*. Twelve of 13 *BolCYCB* genes and 13 of 15 *BraCYCB* genes were in collinear regions. The results showed that segmental duplication was the main contributor in the evolution of *CYCB* members.

To explore the selective pressures on *CYCB* genes, we determined the *Ka*/*Ks* ratio in *Brassica*. The results showed that the *Ka*/*Ks* ratio of all genes were subject to purifying selection. A comparison of the *Ka*/*Ks* ratios showed that the average *Ka*/*Ks* ratio of *B*. *rapa* (0.2064) was higher than that in *B*. *napus* (0.1664) and *B*. *oleracea* (0.1667), suggesting that *CYCB* genes in *B*. *rapa* experienced higher selection pressure during evolution (Table 3).

### 2.5. Analysis of Promoter Cis-Acting Elements and Protein Interaction Networks

**Figure 4:**
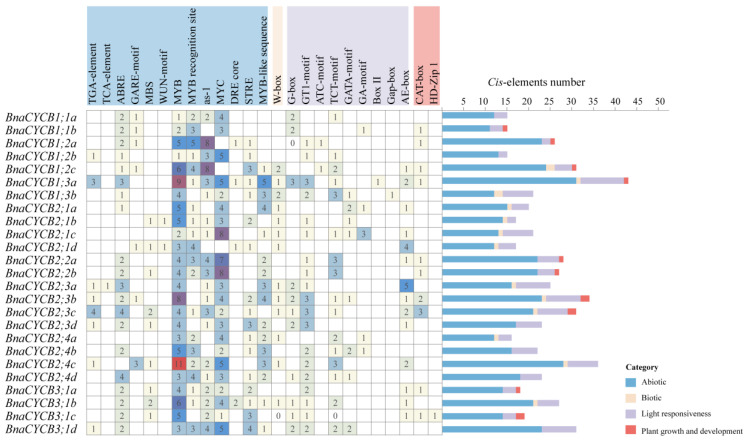
Summary of *cis*-acting elements in the *BnaCYCB* promoter regions.

*Cis*-acting elements affected gene transcription and expression. The analysis showed that the promoter regions of *CYCB* genes contained a lot of *cis*-acting elements involving various biological processes. We excluded the components with general transcriptional regulatory elements (TATA-box, CAAT-box, AT~TATA-box), non-Brassicaceae elements, and uncertain function elements and then divided the remained elements into four categories: abiotic, biotic, light response, and growth and development elements. The abiotic response category was the largest, in which drought stress-responsive elements (MBS, MYB, MYC, MYB recognition site, MYB recognition site) were major and were found in most genes, revealing that *BnaCYCB* genes may be extremely sensitive to drought (Figure 4). Most *BnaCYCB* genes’ promoter regions contained hormone response elements, including abscisic acid (20), auxin (7), gibberellin A3 (7), and ethylene response elements (2). In addition, there was a major proportion of light response elements in *BnaCYCB* gene promoter regions (G-box, GT1-motif, ATC-motif, TCT-motif, GATA-motif, GA-motif, Box-II, Gap-box, AE-box) (Figure 4), suggesting the important role of *BnaCYCB* genes in *B. napus* growth.

**Figure 5:**
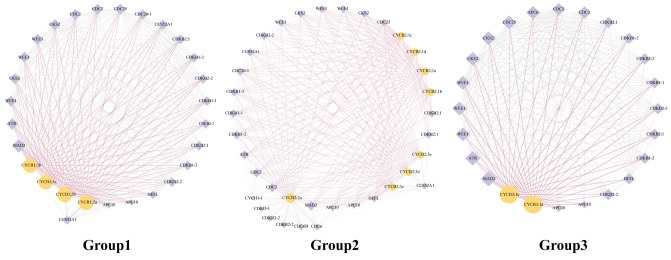
Protein networks for BnaCYCB in Groups 1, 2, and 3. Yellow circles indicate CYCB members, and purple diamonds indicate candidate proteins. The size of the icons represents the degree of the network. Purple lines represent the adjacent edges of CYCB members.

The protein networks displayed that 14 BnaCYCBs potentially interacted with 29 protein candidates (Figure 5), which were mainly associated with cell cycle regulation. The CYCB members from Groups 1, 2, and 3 appeared to display similar networks, showing high connectivity with the hub gene mitotic arrest deficient 2 (*MAD2*) to regulate the spindle assembly checkpoint, which corresponds with their roles in regulating spindle formation (Figure 5). The CYCB proteins were predicted to interact with several types of CDK proteins and some regulatory subunits of CDK (CKS2, WEE1) that may mediate the interactions between CDK and CYCB proteins.

### 2.6. Expression Patterns of BnaCYCB

**Figure 6:**
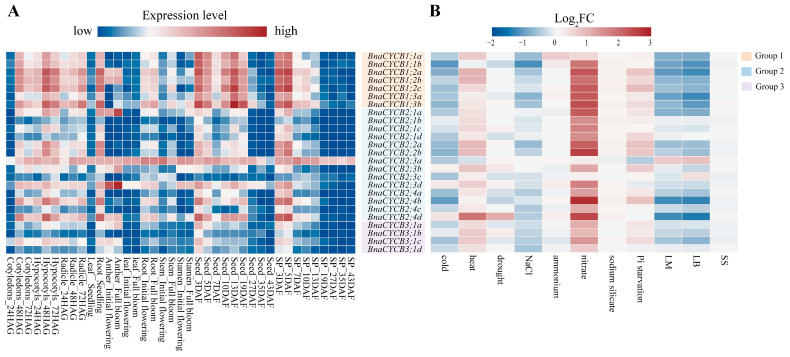
(**A**) Expression patterns of *BnaCYCB* in different organs and development stages. The gene expression level is shown on a graded color scale according to Log2 (FPKM+1) values. HAG, hours after germination; DAF, days after flowering; SP, silique pericarp. (**B**) Expression profiles of *BnaCYCB* in response to abiotic and biotic stress. Gene expression is represented by the Log2 fold change (log2 FC) of the mean (FPKM+1) between the treatment and control. LM, *Leptosphaeria maculans*; LB, *Leptosphaeria biglobosa*; SS, *Sclerotinia sclerotiorum*. The red bar represents high expression, while blue represents little or no expression.

To investigate the tissue-specific expression profiles of *BnaCYCB* genes, we analyzed 10 types of organs at different developmental stages. The results showed that *BnaCYCB* genes tended to show similar expression patterns, with a few exceptions. In the germination stage, most *CYCB* genes were highly expressed in cotyledons, hypocotyls, radicles, and roots, but most *CYCB* members were expressed strongly in the developing seed and silique pericarp in the maturity period (Figure 6A, Table S3). Interestingly, members in Group 1 were more highly expressed compared with Groups 2 and 3, and *BnaCYCB2;3a* was highly expressed in all examined tissues (Figure 6A). Furthermore, we analyzed the expression profiles of *BnaCYCBs* in response to abiotic and biotic stress. The results showed that most genes were sensitive to biotic stress, and their expressions were repressed under cold, drought, NaCl, and ammonium stress (Figure 6B, Table S4), which corresponds with the *cis*-acting element analysis.

### 2.7. Functional Characterization of CYCB3;1 in Mutant A. thaliana

**Figure 7:**
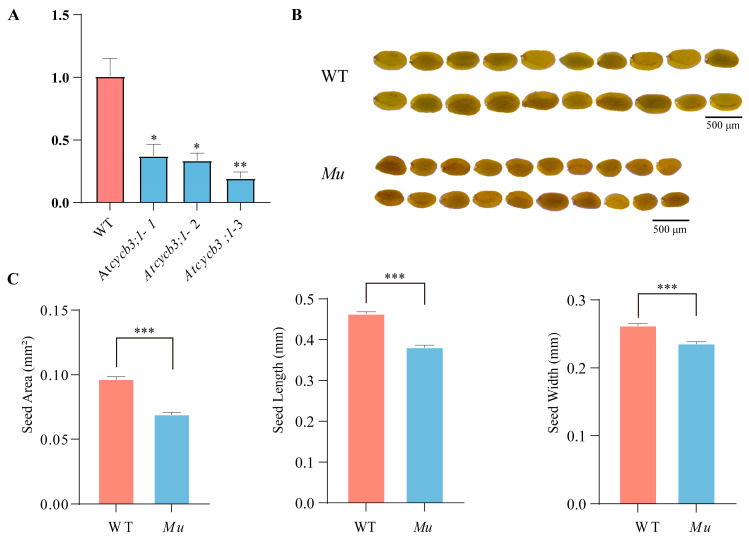
Phenotypes of Col-0 and *Atcycb3;1-Mu* mutant *Arabidopsis* plants. (**A**) Relative normalized expression of *Atcycb3;1-Mu* mutants, * represents significant difference at the 0.05 probability level, ** represents significant difference at the 0.01 probability level; (**B**) comparison of seed size between Col-0 and *Atcycb3;1-Mu*; (**C**) statistical analysis of seed area, seed length, and seed width, *** represents significant difference at the 0.001 probability level. WT refers to wild-type Col-0 plants; *Mu* refers to *Arabidopsis thaliana* mutant *Atcycb3;1-Mu*.

To understand the function of *CYCB3;1*, we examined the phenotypes of T-DNA mutant (*Atcycb3;1-Mu*) in *A. thaliana*. Compared with the wild type (Col-0), *Atcycb3;1-Mu* showed the lower expression level (Figure 7A) and reduced seed size (Figure 7B). Specifically, the seed area, seed length and seed width were significantly lower than those of Col-0 plants (*p* < 0.001) (Figure 7C, Table S5). Moreover, *Atcycb3;1-Mu* did not exhibit obvious growth defects compared to Col-0 plants (Figure S1). These results suggested that *CYCB3;1* is a positive regulator for seed growth.

## 3. Discussion

### 3.1. Characterization of the CYCB Gene Family

We identified 658 *CYCB* genes in 24 angiosperms. Consistent with previous studies [6,23,24], the plant *CYCB* genes could be separated into three groups, with all species having the fewest members in Group 3. We observed that the expansion and shrinkage of *CYCB* members occurred in terms of copy-number within *B*. *napus* accessions and among angiosperm species, corresponding with previous studies [24,25,26]. Some *CYCB* genes have been described previously in maize, soybean, and rice [6,24,27], but several new *CYCB* genes were identified in this study, possibly due to the different identification methods and accession genome sequences used. With a few exceptions, the orthologous genes within each subfamily shared similar gene structures and conserved protein motifs, but great differences were observed among subfamilies, implying functional conservation among homologous genes, as described in other species [24,25,26]. Although plants encode more CDKs and cyclins to interact together to form a potentially great number of combinations [1], the CYCB1 group forms the most active complexes with CDKB2;2 [19]. Group 3 actively binds with CDKB1;1, and Group 2 binds with CDKB1;2 [9], possibly due to the diversity in protein structures among subfamilies. Several studies have confirmed that MSA *cis*-acting elements regulate G2/M transcription in plants [21,28,29]. However, we did not predict MSA elements in this study, possibly because of the strict standards regarding the deletion of MSA elements from non-Brassicaceae species.

### 3.2. Evolutionary Relationships of CYCB Proteins

The CYCB1 and CYCB2 groups were presented before the split of monocots and eudicots and existed before the origin of seed plants [5]. This corresponds with our study, which showed that the CYCB1 and CYCB2 groups have representatives in both monocot and eudicot species. Interestingly, we found that Group 3 members only exist in eudicots, and there were only two ancient copies of *CYCB* (in Groups 1 and 2) presented in *A. trichopoda*−−the common ancestor of both monocots and eudicots. We speculate that an additional *CYCB* copy evolved in eudicots from Group 1, and two ancient *CYCB* copies were retained and evolved through gene duplication, resulting in the three extant groups in the examined eudicot plants (Figure 1C). A previous study reported that *CYCB3;1* is the only B-type cyclin expressed in meiosis, and its proteins interacted together with SDS to control cell wall metabolism in pollen mother cells [30]. Similar to seed development processes in which *Arabidopsis* expresses more genes than barley [31], eudicots may express a more complex regulatory network relating to the evolution of *CYCB3;1* in order to adapt to complex machinery. For example, pollen mother cells are nearly round and are large in volume in monocot plants but polygonal and more complex in eudicot plants [32,33]. In general, the three *Brassica* species showed a strong collinear relationship, because *Brassica* species had undergone whole-genome triplication (WGT), and *B*. *napus* was generated by the progenitor species of *B*. *rapa* and *B*. *oleracea* [34]. We think that WGT and duplications, along with the gene loss that occurred in some copies, may play an important role in *CYCB* evolution from two ancient copies in *A. trichopoda* to 25 copies in *B*. *napus*.

### 3.3. Expression Profile of CYCB Genes

The spatiotemporal expression pattern of genes can provide insight into potential functions. In this study, *CYCB* exhibited tissue- and stage-specific expression, which is in accordance with other studies [24,30]. The *CYCB* genes showed higher expression in the seedling period and rapidly proliferating organs (e.g., developing seed and silique pericarp), agreeing with their essential roles in mitotic cell cycle and/or mitotic growth [35]. Unlike in animals, plant organ size is largely influenced by cell number and depends on the rate of cell division, the number of dividing cells, and the duration of the division phase [16,36]. Different cell cycle types influence organ development and size. The link between cell size and cell cycle progression has been demonstrated in *Arabidopsis* and yeast [37,38,39]. Endoreduplication can enlarge plant cells up to hundreds or even thousands of times compared with the original size [10,11], while asymmetric cell divisions generate different cell fates [40]. Consistent with a previous study, the *cycb3;1* mutants did not show defects during developing periods [30], but did exhibit significantly reduced seed sizes (*p* < 0.001), suggesting potential vales in increasing seed size. The knockdown of *CYCB1;1* in rice likely results in the production of abnormal seeds containing only an enlarged embryo at maturity [41]. However, Group 3 members were downregulated compared with the other groups in this study, despite a report that Group 3 members are relatively conserved and play an important role in animals [42], possibly because they are more important for regulating meiosis than mitosis [43,44,45].

Salinity, moisture, and temperature are well-known factors influencing crops growth and crop yield as a result of cell proliferation and cell expansion. It has been proved that salt, drought, and cold stress severely decrease the expression of cyclins [46,47,48], which confirmed our results of the declined expression of *BnaCYCB* under stress and a great proportion of abiotic elements in their promoter regions. Moreover, a previous study suggested that plants could simultaneously suppress the expression of regulatory genes in the cell cycle under stress conditions [47]. The expression level of *CYCB1;2* promoter activity is transiently decreased under 0.5% NaCl treatment in *A. thaliana* [49]. In rice, cold treatment suppresses the expression of *OsCycB1;1*, *OsCycB2;1,* and *OsCycB2;2*, but overexpressing *OsCycB1;1* plants enhance resistance to cold stress [50]. Additionally, B-type cyclins show downregulation when being treated with SMP values of −309.9 kPa in rice [47]. We believe that plants have evolved a self-protecting mechanism though suppressing the expression level of cyclins under abiotic stress. 

## 4. Materials and Methods

### 4.1. Data Resources

Genomic, coding, and proteomic sequences of *A. thaliana* and all *B*. *napus* accessions (including Dar, Express617, Gangan, Ningyou, NO.2127, Quinta, Shengli, Tapidor, Westar, Zheyou, and ZS11) were obtained from the *Arabidopsis* Information Resource (TAIR, http://www.arabidopsis.org, accessed on 23 November 2023) and the *Brassica napus* multi-omics information resource (BnIR, http://yanglab.hzau.edu.cn/BnIR, accessed on 23 November 2023), respectively. The sequences from *B*. *rapa* and *B*. *oleracea* were downloaded from the Brassicaceae Database (BRAD, http://brassicadb.org/brad, accessed on 28 November 2023). Those from *Solanum lycopersicum*, *A. trichopoda*, *Gossypium raimondii*, *Glycine max*, *O. sativa*, *Zea mays*, *Sorghum bicolor*, *Setaria italica*, *Solanum tuberosum*, *Malus domestica*, *L. usitatissimum*, *Spinacia oleracea*, *Triticum aestivum*, *Saccharum officinarum*, *M. acuminata*, *A. comosus*, *Setaria viridis*, and *Panicum virgatum* were retrieved from Phytozome 13.0 (https://phytozome-next.jgi.doe.gov, accessed on 19 February 2024), and those from *Nicotiana tabacum* and *Capsicum annuum* were retrieved from the Solanaceae Genomics Network (https://solgenomics.net/, accessed on 19 February 2024).

### 4.2. Identification of CYCB Genes

The *CYCB* genes in species were first retrieved using a reciprocal Basic Local Alignment Search Tool Protein (BLASTP) [51,52] at a threshold E-value of 1 × 10^−5^ and a minimum alignment coverage of 50% using 11 *Arabidopsis* CYCB proteins as query sequences [5]. Then, all filtered candidate proteins were used as queries using BLASTP analysis to search against the *A. thaliana* proteome database to investigate their corresponding orthologs at the threshold and minimum alignment coverage parameters described above. To accurately identify genes, all protein sequences were further analyzed in PfamScan (http://www.ebi.ac.uk/Tools/pfa/pfamscan/, accessed on 23 November 2023) to confirm the presence of cyclin-N domains. We named them according to homologous genes in *A. thaliana*.

### 4.3. Protein Sequence Analysis

The amino acids, protein molecular weight, theoretical isoelectric point, and average of hydropathicity were predicted using the ProtParam tool in ExPASy (http://web.expasy.org/protparam/, accessed on 1 December 2023) [53]. The TMHMM-2.0 tool (https://services.healthtech.dtu.dk/services/TMHMM-2.0/, accessed on 1 December 2023) [54] was used to predict the transmembrane transport peptides, and SignalP5.0 (http://www.cbs.dtu.dk/services/SignalP/, accessed on 1 December 2023) [55] was used to identify signal peptides, with default parameters. The subcellular locations of each protein were predicted with Plant-mPLoc (http://www.csbio.sjtu.edu.cn/bioinf/plant-multi/, accessed on 1 December 2023) [56] using default parameters.

### 4.4. Phylogenetic and Evolutionary Analysis

Multiple sequence alignment of CYCB protein sequences was conducted using Molecular Evolutionary Genetics Analysis (MUSCLE, University of Michigan, Ann Arbor, MI, USA) with default parameters [57]. Then, neighbor-joining (NJ) trees were constructed using Molecular Evolutionary Genetics Analysis11 (MEGA11, Tokyo Metropolitan University, Tokyo, Japan) [58], with the following parameters: p-distance + G substitution model and 1000 bootstrap replications.

To further understand the evolutionary relationships of the gene families, the collinearity information among *A. thaliana* and Brassicaceae species was obtained using the Multiple Collinearity Scan toolkit (MCScanX) [59] using the following parameters: match_score: MATCH_SIZE: 5, gap_penalty: −1, overlap_window: 5, E_value: 1 × 10^−5^, max_gaps: 25. Then, the CDS and protein sequences were used to calculate the synonymous mutation rate (*Ks*), non-synonymous mutation rate (*Ka*), and evolutionary constraint (*Ka*/*Ks*) in TBtools v2.082 (South China Agricultural University, Guangzhou, China) [60], where *Ka*/*Ks* < 1 indicates purifying selection, *Ka*/*Ks* = 1 indicates neutral selection, and *Ka*/*Ks* > 1 indicates positive selection.

### 4.5. Gene Structure, Protein Motif Identification, and Chromosomal Location Analysis

The Gene Structure Display Server (GSDS 2.0: https://gsds.gao-lab.org/, accessed on 1 December 2023) [61] was used to determine the exon/intron structures of the *CYCB* genes. The Multiple Expectation Maximization for Motif Elicitation program (MEME 4.12.0, https://meme-suite.org/meme/doc/download.html, accessed on 1 December 2023) [62] was used to identify conserved motifs in CYCB proteins from *B*. *napus* with the following parameter settings: the minimal and maximal motif widths were set to 6 and 200 amino acids, respectively, and the number of motifs was 15. Only motifs with an *e*-value of <1 × 10^−10^ were kept for further motif analysis (other parameters were set to default). Finally, the results of gene structure, protein motif, and chromosomal location were visualized using TBtools v2.082 [60].

### 4.6. Analysis of Promoter Cis-Acting Elements and Protein Interaction Networks

The 2000 bp upstream sequences of *CYCB* genes initiation codon were obtained with TBtools software v2.082 [60] and used for the prediction of *cis*-acting elements using the online tool PlantCARE (https://bioinformatics.psb.ugent.be/webtools/plantcare/html/, accessed on 12 January 2024) [63]. The STRING 12.0 database (http://www.string-db.org, accessed on 31 March 2024) [64] was used to investigate the interaction networks of 14 CYCB proteins, with a minimum required interaction score of 0.7, and the network was ranked using the cytoHubba plugin [65] and visualized by Cytoscape 3.7.0 (Institute for Systems Biology, Seattle, WA, USA) [66].

### 4.7. Expression Patterns in Tissues and Under Stress

The expression data of 10 tissue samples and under 11 biotic and abiotic stress were obtained from the BrassicaEDB database (https://biodb.swu.edu.cn/brassica/, accessed on 12 January 2024) [67] to explore the expression patterns of the *CYCB* genes. The tissues included the cotyledons, hypocotyls, and germinating radicle after 24, 48, and 72 h; the leaf and root in the seedling stage, initial flowering, and full bloom stage; the anther, stem, and stamen in the initial flowering stage and full bloom stage; and the seeds, silique pericarp, and flowers after 3, 5, 7, 10, 13, 19, 27, 35, and 43 days. The stresses included cold, heat, drought, NaCl, ammonium, nitrate, sodium silicate, Pi starvation, *Leptosphaeria maculans*, *Leptosphaeria biglobosa*, and *Sclerotinia sclerotiorum*. The tissue expression levels of the *CYCBs* were normalized by Log_2_ (FPKM+1), and stress expression was normalized using the Log_2_ fold change (the mean (FPKM+1) between the treatment and control), and heatmaps were visualized using Rstudio.

### 4.8. Plant Material and Culture Conditions

Seeds of *A. thaliana* ecotype Col-0 and *AtCYCB3;1* T-DNA insertion mutant (*Atcycb3;1-Mu*) were obtained from AraShare (https://www.arashare.cn, accessed on 21 November 2023) and were cultivated in Southwest University, Chongqing, China (29°49′18″ N, 10°25′45″ E). All *Arabidopsis* seeds were firstly sown on half-strength Murashige and Skoog (MS) medium and then transplanted to a growth chamber using a peat-based soil mixture, Pindstrup 2 (Pindstrup, Ryomgaard, Denmark) when the fourth leaf emerged, where it was environmentally controlled with a light period from 8:00 a.m. to 10:00 p.m. at 22 °C, a dark period from 10:00 p.m. to 8:00 a.m. at 18 °C, 60% relative humidity, and 1.1 × 10^−6^ μmol m^−2^ s^−1^ light intensity.

### 4.9. DNA and RNA Extraction and Phenotypic Observation 

The 21-day-old leaves of *Atcycb3;1-Mu* plants were collected for DNA extraction using the CTAB method [68], and then homozygous *AtCYCB3;1-Mu* mutants were screened and identified using the primers *CYCB3;1* LP/LBb1.3 and LBb1.3/*CYCB3;1* RP (Table S6). Total RNA was extracted from the leaves of 30-day-old seedlings of *Arabidopsis* Col-0 wildtype and *Atcycb3;1-Mu*, and first-strand cDNA was synthesized using a HiScript III RT SuperMix for qPCR kit (Vazyme, Nanjing, China) according to the manufacturer’s instructions. Quantitative real-time (qRT)-PCR reactions were performed as described in the MIQE guidelines [69] (Table S6), with three technical replicates for each sample. Relative expression levels were calculated using the 2^∆∆Ct^ method, with *Actin7* as the internal reference gene. The seed area, seed length and seed width were measured using ImageJ v1.53t software (National Institutes of Health, Bethesda, MD, USA). 

### 4.10. Statistical Analysis

Values were expressed as means ± standard error, and statistically significant differences were determined using Student’s *t*-test: *, *p* < 0.05; **, *p* < 0.01; ***, *p* < 0.001. The results were displayed using GraphPad Prism 9.5 software (GraphPad Software, San Diego, CA, USA).

## 5. Conclusions

In this study, we identified and characterized *CYCB* family members in *B. napus* and evaluated the phylogenetic relationship of *CYCB* genes from 24 angiosperm species. Collinearity analysis suggested that *BnaCYCB* exhibited segmental duplication. The *BnaCYCB* genes tended to show higher expression levels in proliferating organs and were downregulated under abiotic stress. The *A. thaliana* mutant *Atcycb3;1-Mu* indicated a positive role of *CYCB* in seed growth.

## Figures and Tables

**Table 1:** Physical and chemical properties of cyclin B proteins in *Brassica napus*.

Gene Name	Homologs in *Arabidopsis*	Amino Acid	Molecular Weight	Isoelectric Point	Average of Hydropathicity	mPLoc
*BnaCYCB1;1a*	*AT4G37490*	428	48,536.74	8.45	−0.404	Nucleus
*BnaCYCB1;1b*		419	47,489.63	6.85	−0.356	Nucleus
*BnaCYCB1;2a*	*AT5G06150*	433	48,504.42	9.36	−0.300	Nucleus
*BnaCYCB1;2b*		681	76,106.19	9.10	−0.280	Nucleus
*BnaCYCB1;2c*		428	48,184.08	9.32	−0.321	Nucleus
*BnaCYCB1;3a*	*AT3G11520*	394	44,243.95	8.11	−0.204	Nucleus
*BnaCYCB1;3b*		207	23,634.39	5.49	0.130	Nucleus
*BnaCYCB2;1a*	*AT2G17620*	419	48,148.57	5.31	−0.297	Nucleus
*BnaCYCB2;1b*		409	46,916.94	4.99	−0.229	Nucleus
*BnaCYCB2;1c*		418	47,976.40	5.53	−0.315	Nucleus
*BnaCYCB2;1d*		407	46,458.54	5.22	−0.226	Nucleus
*BnaCYCB2;2a*	*AT4G35620*	406	46,807.49	4.81	−0.333	Nucleus
*BnaCYCB2;2b*		401	46,228.81	4.86	−0.346	Nucleus
*BnaCYCB2;3a*	*AT1G20610*	410	46,983.71	5.24	−0.345	Nucleus
*BnaCYCB2;3b*		410	46,687.53	5.15	−0.367	Nucleus
*BnaCYCB2;3c*		232	26,544.13	9.11	0.016	Nucleus
*BnaCYCB2;3d*		408	46,435.24	5.31	−0.354	Nucleus
*BnaCYCB2;4a*	*AT1G76310*	413	47,568.57	5.40	−0.397	Nucleus
*BnaCYCB2;4b*		431	49,164.17	5.06	−0.413	Nucleus
*BnaCYCB2;4c*		408	46,918.93	5.38	−0.317	Nucleus
*BnaCYCB2;4d*		438	50,147.33	5.14	−0.410	Nucleus
*BnaCYCB3;1a*	*AT1G16330*	609	68,343.75	9.81	−0.446	Nucleus
*BnaCYCB3;1b*		204	23,927.39	9.50	−0.113	Nucleus
*BnaCYCB3;1c*		584	65,353.07	9.84	−0.528	Nucleus
*BnaCYCB3;1d*		588	66,241.50	9.86	−0.469	Nucleus

**Table 2:** Copy-number variations in *CYCB* members in 11 *Brassica napus* accessions.

Accession	Total	*CYCB1;1*	*CYCB1;2*	*CYCB1;3*	*CYCB1;4*	*CYCB1;5*	*CYCB2;1*	*CYCB2;2*	*CYCB2;3*	*CYCB2;4*	*CYCB3;1*
Dar	25	2	3	2	0	0	4	2	4	4	4
Express617	27	2	3	3	4	2	4	5	4	0	0
Gangan	25	1	4	2	4	2	5	2	5	0	0
Ningyou	50	8	10	4	4	4	4	7	9	0	0
NO.2127	30	1	4	3	4	3	4	7	4	0	0
Quinta	24	0	4	2	4	2	4	4	4	0	0
Shengli	24	1	3	3	3	2	4	4	4	0	0
Tapidor	27	3	1	3	4	3	4	5	4	0	0
Westar	25	1	3	3	4	2	4	4	4	0	0
Zheyou	22	0	2	3	4	2	4	4	3	0	0
ZS11	20	0	3	3	0	0	4	2	4	0	4

**Table 3:** Nucleotide substitution rates of *Brassica napus*, *Brassica rapa,* and *Brassica oleracea*.

Species	Name	*Ka*	*Ks*	*Ka*/*Ks*
	*BnaCYCB1;1a*	0.0840	0.4688	0.1791
	*BnaCYCB1;1b*	0.0823	0.4923	0.1672
	*BnaCYCB1;2a*	0.0826	0.7738	0.1067
	*BnaCYCB1;2b*	0.0756	0.9037	0.0837
	*BnaCYCB1;2c*	0.0801	0.7714	0.1038
	*BnaCYCB1;3a*	0.1018	0.6143	0.1658
	*BnaCYCB1;3b*	0.1527	0.7007	0.2179
	*BnaCYCB2;1a*	0.0733	0.4211	0.1741
	*BnaCYCB2;1b*	0.0886	0.4631	0.1912
	*BnaCYCB2;1c*	0.0660	0.4304	0.1534
	*BnaCYCB2;1d*	0.0872	0.4414	0.1975
*Brassica napus*	*BnaCYCB2;2a*	0.0607	0.3799	0.1598
	*BnaCYCB2;2b*	0.0589	0.4353	0.1354
	*BnaCYCB2;3a*	0.1112	0.4006	0.2775
	*BnaCYCB2;3b*	0.0774	0.4841	0.1599
	*BnaCYCB2;3c*	0.0427	0.4475	0.0955
	*BnaCYCB2;3d*	0.0781	0.4388	0.1779
	*BnaCYCB2;4a*	0.0672	0.3856	0.1743
	*BnaCYCB2;4b*	0.0686	0.4981	0.1377
	*BnaCYCB2;4c*	0.0761	0.4487	0.1695
	*BnaCYCB2;4d*	0.0670	0.4529	0.1479
	*BnaCYCB3;1a*	0.1117	0.4301	0.2596
	*BnaCYCB3;1b*	0.0402	0.6506	0.0618
	*BnaCYCB3;1c*	0.0920	0.4055	0.2269
	*BnaCYCB3;1d*	0.1025	0.4343	0.2360
	*BraCYCB1;1a*	0.2335	0.7303	0.3197
	*BraCYCB1;1b*	0.2386	0.7516	0.3174
	*BraCYCB1;1c*	0.0806	0.4659	0.1731
	*BraCYCB1;2a*	0.0749	0.7706	0.0973
	*BraCYCB1;2b*	0.0715	0.7975	0.0897
	*BraCYCB1;3a*	0.1323	0.6803	0.1945
	*BraCYCB2;1a*	0.0768	0.4280	0.1793
*Brassica rapa*	*BraCYCB2;1b*	0.0885	0.4104	0.2157
	*BraCYCB2;2a*	0.0631	0.3669	0.1719
	*BraCYCB2;3a*	0.1142	0.3730	0.3063
	*BraCYCB2;3b*	0.0774	0.4846	0.1598
	*BraCYCB2;4a*	0.1031	0.4352	0.2369
	*BraCYCB2;4b*	0.0660	0.4687	0.1408
	*BraCYCB3;1a*	0.1078	0.4193	0.2572
	*BraCYCB3;1b*	0.1095	0.4630	0.2366
	*BolCYCB1;1a*	0.1099	0.4416	0.2489
	*BolCYCB1;3a*	0.0747	0.8366	0.0893
	*BolCYCB1;2a*	0.0883	0.7987	0.1106
	*BolCYCB1;2b*	0.1101	0.6067	0.1815
	*BolCYCB2;1a*	0.0660	0.4304	0.1534
	*BolCYCB3;1a*	0.0860	0.4484	0.1918
*Brassica oleracea*	*BolCYCB2;3a*	0.0584	0.4331	0.1349
	*BolCYCB2;4a*	0.0433	0.4574	0.0948
	*BolCYCB2;4b*	0.0782	0.4567	0.1711
	*BolCYCB2;2a*	0.0737	0.4563	0.1615
	*BolCYCB3;1b*	0.0670	0.4407	0.1519
	*BolCYCB2;3b*	0.0913	0.4115	0.2220
	*BolCYCB2;1b*	0.1083	0.4242	0.2552

## Data Availability

All data are contained within the article and its Supplementary Materials.

## Supplementary Materials

The following supporting information can be downloaded at: https://www.mdpi.com/article/10.3390/plants13121709/s1, Figure S1: Phenotypic observation of *Arabidopsis thaliana* mutant *cycb3;1*; Table S1: CYCB protein information in Brassicaceae; Table S2: CYCB protein information in 20 angiosperms; Table S3: FPKM values of *BnaCYCB* genes in 10 types of organs; Table S4: FC values (the mean (FPKM+1) between the treatment and control) of *BnaCYCB* genes under abiotic and biotic stress; Table S5: Figures for seed area, seed length, and seed width; Table S6: Primers used in this study.
