# Supplementary material for: Genome-Wide Identification and Evolutionary and Expression Analyses of the Cyclin B Gene Family in Brassica napus
Source: Plants (Basel). 2024 Jun 20;13(12):1709. doi: 10.3390/plants13121709 (PMC11207893; doi:10.3390/plants13121709)
Supplement: Supplementary file 1 [file plants-13-01709-s001.zip › Supplementary figure.pdf]

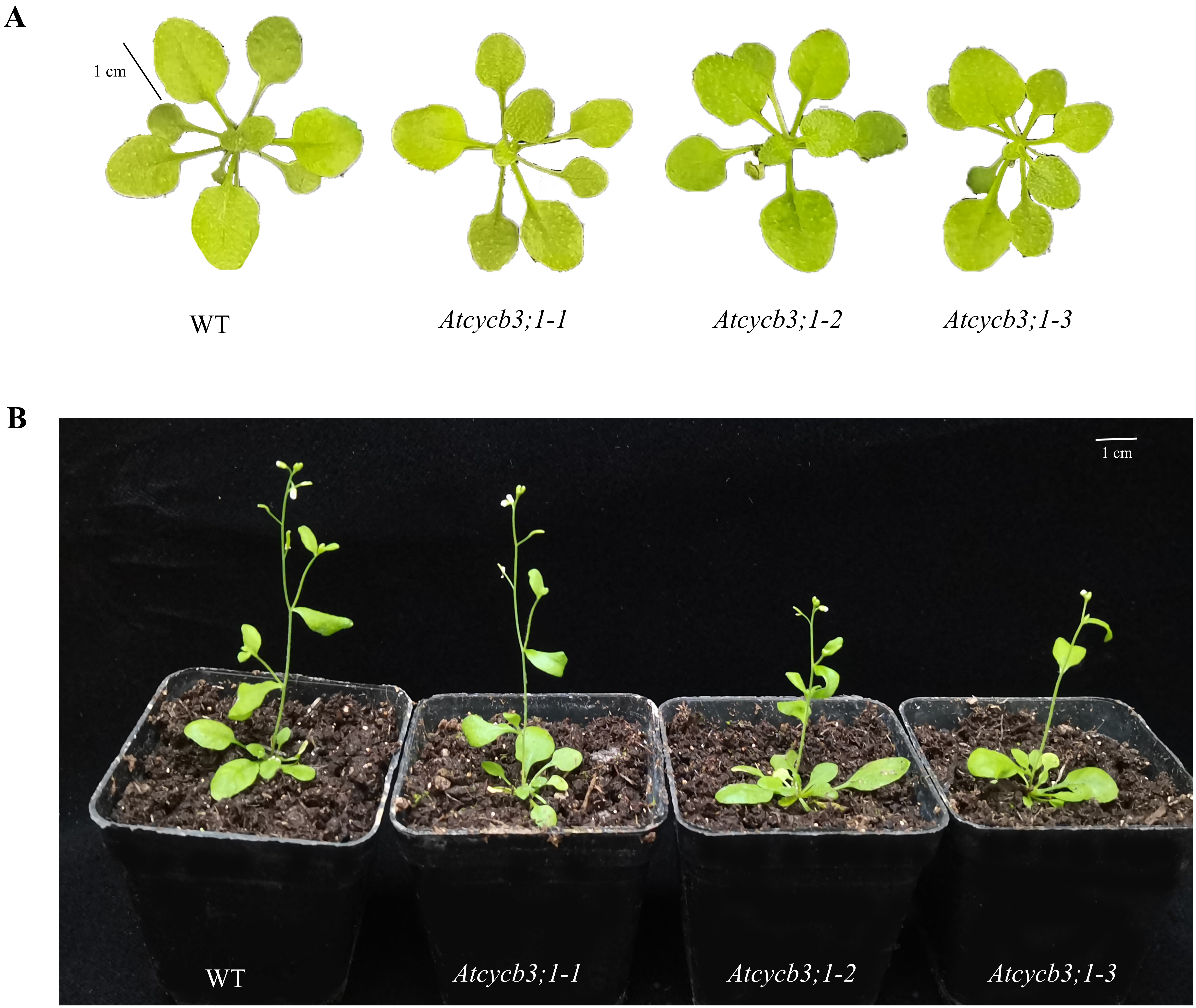

Figure S1. Phenotypic observation of *Arabidopsis thaliana* mutant *cycb3;1*. (A)Observation of 28 day-old seedlings of *Arabidopsis* Col-0 wildtype and *Atcycb3;1-Mu*; (B)Observation of 42 day-old seedlings of *Arabidopsis* Col-0 wildtype and *Atcycb3;1-Mu*. WT refers to wild-type Col-0 plants.
